# Anthropometric correlation with hamstring graft size in anterior cruciate ligament reconstruction among males

**DOI:** 10.1007/s00264-019-04452-5

**Published:** 2019-12-26

**Authors:** Isam Moghamis, Yousef Abuodeh, Ali Darwiche, Talal Ibrahim, Mohammad Al Ateeq Al Dosari, Ghalib Ahmed

**Affiliations:** 1 Orthopedics Department, Hamad General Hospital, Hamad Medical Corporation, PO Box 3050, Doha, Qatar; 2 Pediatric Orthopedic, SIDRA Hospital, Doha, Qatar; 3 Weil Cornell Medical College, Ar-Rayyan, Qatar

**Keywords:** Anterior cruciate ligament, Hamstring graft size, Anthropometric measurements

## Abstract

**Purpose:**

Pre-operative knowledge of hamstring graft size for anterior cruciate ligament reconstruction (ACL) is of clinical importance and useful in making appropriate decisions about graft choice. This study investigated if there is any correlation between anthropometric measurements such as height, weight, body mass index, thigh length, and circumference with the size of hamstring tendon graft in anterior cruciate ligament reconstruction.

**Methods:**

The anthropometric data of 50 consecutive adult males, who underwent primary ACL reconstruction using quadruple hamstring autograft, were collected prospectively. Data analysis using Pearson’s correlation test was performed and multiple logistic regression analysis was used to investigate any correlation not detected by Pearson’s test and to eliminate confounders.

**Results:**

Patient’s height and thigh length demonstrated a positive correlation with gracilis graft length (*r* = .464, *P* = .001, *r* = .456, *P* = .001, respectively) and semitendinosus graft length (*r* = .541, *P* = 000, *r* = .578, *P* = .000, respectively). While the patient’s age was the only independent factor which had a positive correlation with the quadrupled hamstring graft diameter (*r* = .412, *P* = .004), multiple regression analysis showed abdominal girth had a significant negative correlation with gracilis (*P* = .04) and semitendinosus (*P* = .006) graft thickness.

**Conclusion:**

This study demonstrated that some anthropometric measurements had a positive correlation with the hamstring graft length and diameter in male patients. Hence**,** these results provide preliminary support for the use of some anthropometric measurements in the preoperative planning and prediction of the hamstring graft length and diameter in anterior cruciate ligament reconstruction.

## Introduction

Rupture of the anterior cruciate ligament (ACL) is one of the most common encountered knee injuries [1]. Deficiency of this ligament can be severely detrimental to high-level athletes or individuals participating in sports [2].

The aim of an ACL reconstruction is to restore the function and biomechanics of the native ligament. Various grafts available for use in the reconstruction of the ACL and the hamstring is one of the most commonly utilized autograft [3, 4]. A graft diameter greater than 8 mm has been recommended by many authors in order to reduce the risk of graft failure [5–7]. There are considerable variations in the size of hamstring tendons between individuals, and hence graft diameter is often unpredictable.

Pre-operative knowledge of the hamstring graft length and diameter is of clinical importance and may assist surgeons in making appropriate and informed decisions about the graft choices which may increase surgeon’s confidence and enhance patient’s evaluation and counseling regarding graft choice [8–10].

Various studies exist in the literature regarding prediction of graft size. However, no consensus has been reached due to differences in results between the studies [11–13].

This study investigated if there is any correlation between anthropometric measurements such as height, weight, body mass index, thigh length, and thigh circumference with the size of hamstring tendon graft in anterior cruciate ligament (ACL) reconstruction.

We hypothesized that there is no correlation between some anthropometric measurements with the size of the hamstring tendon graft that is used in anterior cruciate ligament reconstruction among males.

## Material and methods

We prospectively collected anthropometric data of 50 consecutive patients with ACL deficiency confirmed by MRI studies pre-operatively and scheduled to undergo primary arthroscopic ACL reconstruction using a single-bundle quadruple hamstring tendon autograft, between September 2014 and January 2017. Approval for the study was obtained from our Institutional Review Board.

All adult male patients with isolated ACL deficiency undergoing primary arthroscopic ACL reconstruction using a hamstring tendon autograft were included in this study. Female patients, children under the age of 18 years, patients who had undergone previous ACL reconstruction, multiple ligamentous injuries, patients treated using grafts other than hamstring, double-bundle hamstring graft reconstructions, single-bundle hamstring graft reconstruction, and patients with neuromuscular diseases were excluded from the study.

Informed consent was obtained from all patients prior to their inclusion. The following data was collected from patients: age, ethnicity, height, weight, abdominal girth, thigh length, and thigh circumference. The abdominal girth, thigh length, and thigh circumference measurements were taken while the patients were supine and knees in full extension.

Abdominal girth was measured by placing the tape around the abdomen at the level of the umbilicus. Thigh length was measured from the anterior superior iliac spine (ASIS) to the superolateral border of the patella. The thigh circumference was measured at a point 15 cm proximal to the superolateral border of the patella.

**Fig. 1:**
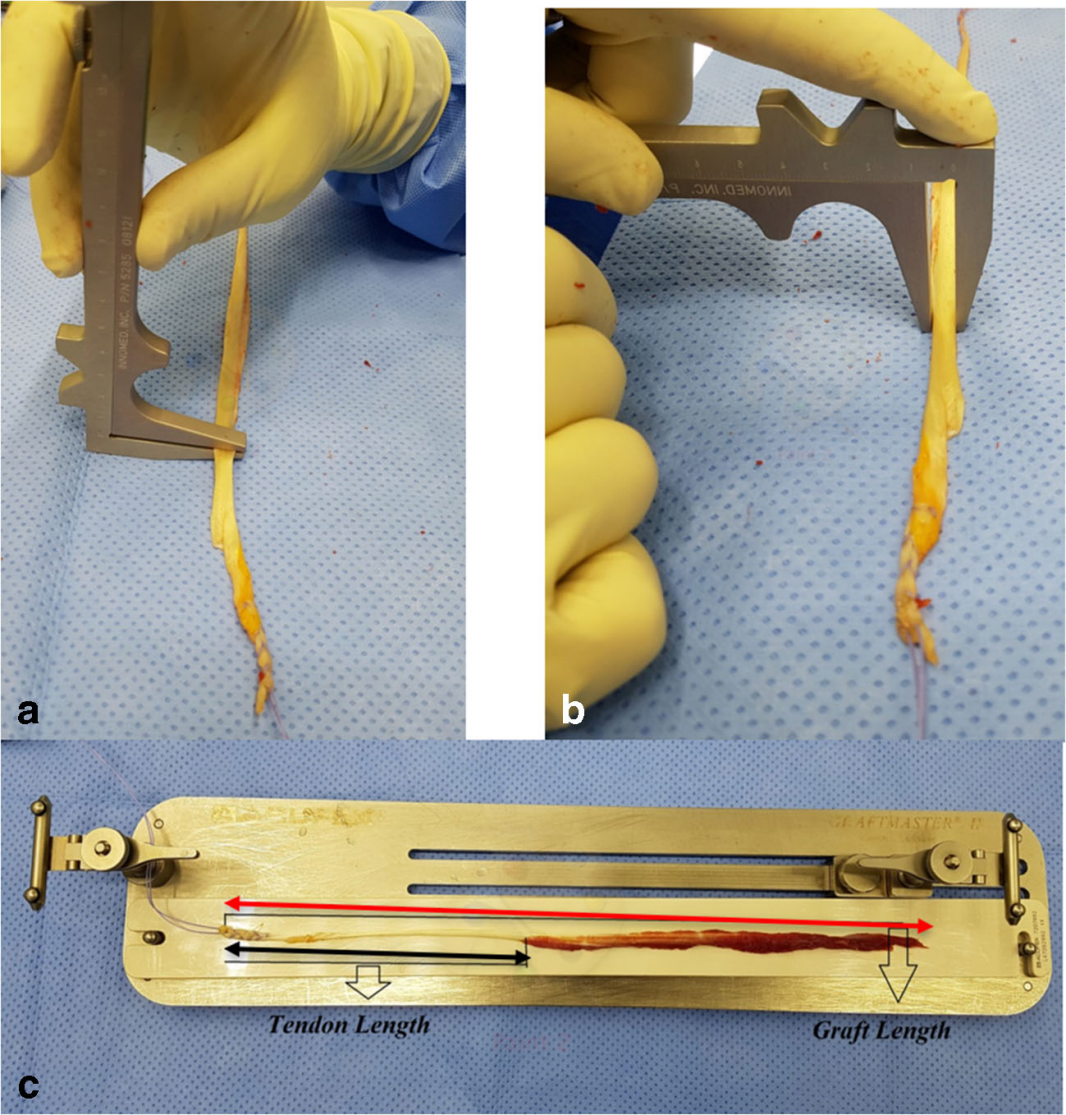
**a** Graft thickness measurement. **b** Graft width measurements. **c** Tendon length (black arrow) and graft length (red arrow) measurements

Two senior board certified fellowship trained knee surgeons performed all operations using the same harvesting technique. Using a skin incision distal to the insertion of the tendons on the proximal tibia, both semitendinosus and gracillis tendons were harvested by a closed graft harvester. Graft length was determined from the tibia insertion including the pretibial periosteum to the tendon tail while tendon length was from the tibia insertion including the pretibial periosteum to the tendomuscular junction. Intraoperative measurements of each tendon were recorded by the operating surgeons after removal of the fat and muscle tissue attached to each tendon. The measurements included length of the full graft, length of tendon, its width, and thickness of the tendons in millimeters (Fig. 1).

**Fig. 2:**
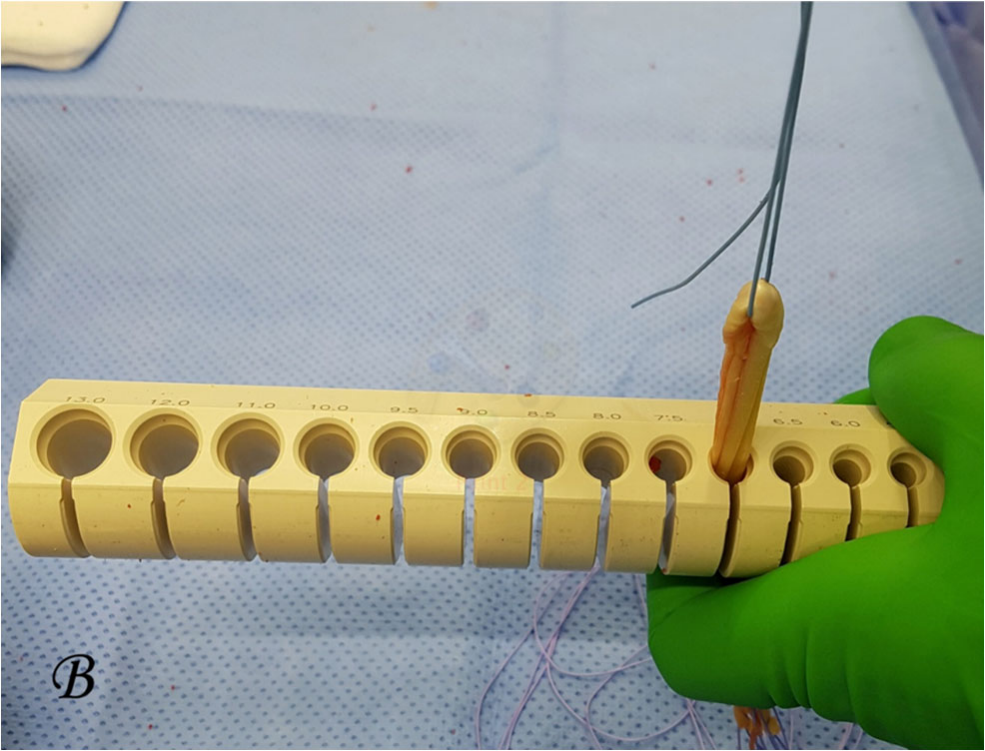
Hamstring graft diameter measurement using a diameter measurement tool (Smith and Nephew, Androver, USA)

The hamstring graft was prepared using a single-bundle 4-strand technique with each end of the tendon whip stitched using the same non-absorbable size 2 ethibond suture. The final graft diameter was measured using the ACL reconstruction graft diameter measurement guide (Smith and Nephew, Androver, USA) and the diameter was defined as the smallest calibrated size in which the graft could pass through (Fig. 2).

### Data analysis

IBM SBSS Statistics (version 24) software was used for statistical analysis. Pearson’s test was used to identify correlations between anthropometric measurements and graft dimension. For results with positive correlation, simple linear regression analysis was used to estimate the linear curves. Multiple logistic regression analysis was used to investigate correlations not detected by Pearson’s test and to eliminate confounders. The positive results were considered statistically significant if the *P* value was less than 5% (*P* ≤ .05).

## Results

A total number of 50 consecutive male patients agreed to participate in the study. There were 3 patients who had missing data of the final graft diameter. Table 1 presents the means of patient’s demographics along with the mean of the anthropometric measurement taken.

**Table 1:** Patients’ characteristics

		Mean	SD
Age (year)		29	7
Weight (kg)		82.2	11.2
Height (cm)		174	8
BMI		27.0	3.5
Abdominal girth (cm)		86.80	8.734
Thigh length (cm)		46.64	2.701
Thigh circumference (cm)		50.74	3.784
Surgery side		*N*	%
	Left	23	46.0
	Right	27	54.0
Measurement side		*N*	%
	Left	26	52.0
	Right	24	48.0
Ethnicity		*N*	%
	African	17	34.0
	Asia	17	34.0
	Middle East	14	28.0
	European	2	4.0

The mean length of the harvested gracilis tendons was 147.3 mm (± 37.1 SD). Whereas, the mean graft length was 273.9 mm (± 33.1 SD) with mean width of 4.7 mm (± 2.1 SD) and mean thickness of 1.8 mm (± .8 SD). On the other hand, the mean length of harvested Semitendinosus tendons was 172.1 mm (± 38.9 SD) and the mean graft length was 307.5 mm (± 31.9 SD) with a mean width of 6.0 mm (± 2.8 SD) and a mean thickness of 2.3 mm (± .98 SD). The whole, single bundle 4-strands, mean graft diameter was 7.3 mm (± .67 SD) (Table 2).

**Table 2:** Graft measurement outcome

	Parameters	*N*	Min.	Max	Mean	SD
Gracilis	Tendon length (mm)	50	100	270	147.30	37.146
	Graft length (mm)	50	180	350	273.90	33.094
	Width (mm)	50	2.0	10.0	4.730	2.1122
	Thickness (mm)	50	.8	5.0	1.766	.7883
Semitendinosus	Tendon length (mm)	50	100	340	171.00	38.914
	Graft length (mm)	50	240	370	307.50	31.916
	Width (mm)	50	2.0	15.0	6.020	2.7811
	Thickness (mm)	50	1.0	4.0	2.266	.9884
ACL hamstring graft diameter (mm)		47	6.0	9.0	7.266	.6745

Analysis of correlation among results showed that patient’s height demonstrated a significant positive moderate correlation with gracilis graft length (*r* = .464, *P* = .001) and semitendinosus graft length (*r* = .541, *P* = .000). In addition, it showed a weak significant positive correlation with semitendinosus tendon length (*r* = .337, *P* = .017) (Table 3).

**Table 3:** Correlation coefficient between intraoperative measurement and anthropometric data

Gracilis								
	Tendon length		Graft length	Width		Thickness		
	Correlation coefficient	*P* value	Correlation coefficient	*P* value	Correlation coefficient	*P* value	Correlation coefficient	*P* value
Age	− .054	.710	.165	.252	.012	.932	− .054	.708
Weight	− .022	.880	.274	.054	− .090	.533	.044	.759
Height	.192	.181	.464	.001	− .104	.474	.010	.944
BMI	− .141	.329	− .025	.862	− .037	.799	.043	.769
Abdominal girth	.127	.380	.072	.618	− .002	.990	− .112	.438
Thigh length	.187	.192	.456	.001	− .025	.866	.066	.649
Thigh circumference	−.148	.305	− .136	.346	− .144	.317	.239	.094
Semitendinosus								
	Tendon length		Graft length	Width		Thickness		
	Correlation coefficient	*P* value	Correlation coefficient	*P* value	Correlation coefficient	*P* value	Correlation coefficient	*P* value
Age	.089	.539	.139	.334	.070	.631	.051	.727
Weight	.113	.437	.258	.070	− .129	.371	.115	.426
Height	.337	.017	.541	.000	− .013	.928	.097	.503
BMI	− .094	.517	− .093	.520	− .140	.333	.057	.694
Abdominal girth	.104	.472	− .090	.532	.005	.971	− .135	.350
Thigh length	.217	.130	.578	.000	− .091	.528	.238	.097
Thigh circumference	− .093	.520	− .203	.157	− .240	.093	.106	.463
ACL hamstring graft diameter								
	Correlation coefficient	*P* value						
Age	.412	.004						
Weight	.166	.265						
Height	.194	.192						
BMI	.046	.757						
Abdominal girth	.051	.732						
Thigh length	.116	.437						
Thigh circumference	− .073	.624						

Thigh length was found to have a significant positive moderate correlation with gracilis graft length (*r* = .456, *P* = .001) and semitendinosus graft length (*r* = .578, *P* = .000).

Furthermore, patient age demonstrated a significant positive moderate correlation with the final graft diameter (*r* = .412, *P* = .004). There was no statistically significant correlation between hamstring graft diameter and the remaining anthropometric measurements.

**Fig. 3:**
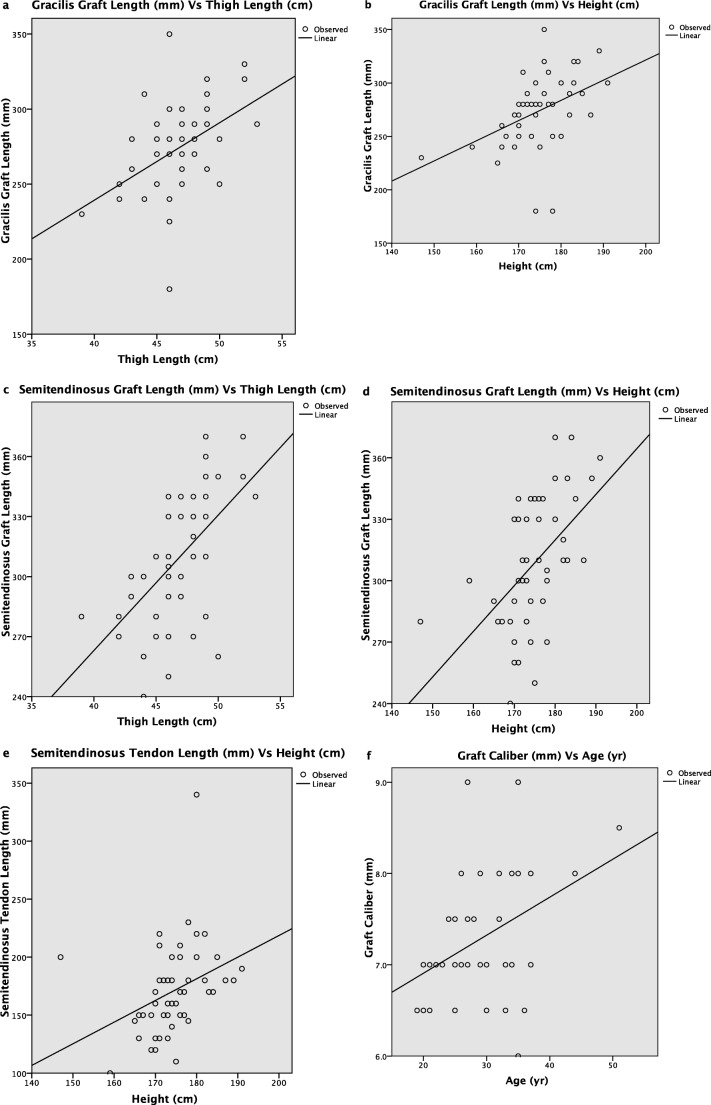
Curve estimates by simple linear regression (*R*-squared values in Table 4)

Simple linear regression analysis results for estimated curves showed that gracilis graft length measurement variation could be explained by patient’s height and thigh length (*R*^2^ = .215, *P* = .001 and *R*^2^ = .207, *P* = .001, respectively). Semitendinosus graft length measurement variations could also be explained by patient’s height and thigh length (*R*^2^ = .292, *P* = .000 and *R*^2^ = .334, *P* = .000, respectively). Semitendinosus tendon length measurement variations were explained by height (*R*^2^ = .114, *P* = .017). Variations of final hamstring graft diameter could be explained by age (*R*^2^ = .170, *P* = .004) (Table 4, Fig. 3).

**Table 4:** Results of simple linear regression analysis

Dependent	Independent	*R*^2^	*P* value
Gracilis graft length	Height	.215	.001
	Thigh length	.207	.001
Semitendinosus graft length	Height	.292	.000
	Thigh length	.334	.000
Semitendinosus tendon length	Height	.114	.017
ACL hamstring graft diameter	Age	.170	.004

Multiple regression analysis demonstrated a significant positive correlation between abdominal girth and gracilis tendon length(*P* = .003) but not significant for semitendinosus tendon length (*P* = .143), a significant negative correlation between abdominal girth and gracilis graft thickness (*P* = .040) and semitendinosus graft thickness (*P* = .006) and a significant positive correlation between the thigh circumference and gracilis graft thickness (*P* = .019) but not significant for semitendinosus graft thickness (*P* = .262), whereas thigh length had a significant positive correlation on only semitendinosus graft length (*P* = .016). The age was found to have a significant positive effect on the final ACL hamstring graft diameter (*P* = .013) (Table 5). Finally, the number of patients in the different ethnic groups was small and any statistical analysis would be misleading.

**Table 5:** Results of multiple regression analysis

	Gracilis							
	Tendon length		Graft length		Width		Thickness	
	Coefficient	*P* value	Coefficient	*P* value	Coefficient	*P* value	Coefficient	*P* value
Constant	− .923	.362	− .028	.978	1.442	.157	− .532	.598
Age	− .625	.535	.943	.351	− .211	.834	.731	.469
Weight	− 1.017	.315	.064	.949	1.215	.231	− .420	.677
Height	.940	.353	.166	.869	− 1.344	.186	.445	.658
BMI	.650	.519	.021	.984	− 1.200	.237	.435	.666
Abdominal girth	3.121	.003	.086	.932	.807	.424	− 2.121	.040
Thigh length	.822	.416	1.562	.126	.406	.687	.587	.560
Thigh circumference	− .597	.554	− .888	.380	− 1.160	.253	2.431	.019
	Semitendinosus							
	Tendon length		Graft length		Width		Thickness	
	Coefficient	*P* value	Coefficient	*P* value	Coefficient	*P* value	Coefficient	*P* value
Constant	− 1.545	.130	− .236	.815	1.722	.092	− .491	.626
Age	.517	.608	1.381	.174	.166	.869	1.151	.256
Weight	− 1.467	.150	− .122	.904	1.496	.142	− .355	.724
Height	1.614	.114	.447	.657	− 1.526	.135	.386	.701
BMI	1.236	.223	.404	.688	− 1.627	.111	.575	.568
Abdominal girth	1.493	.143	− 1.873	.068	1.786	.081	− 2.882	.006
Thigh length	.169	.867	2.499	.016	− .566	.574	1.601	.117
Thigh circumference	− .025	.981	− 1.099	.278	− 1.278	.208	1.138	.262
	ACL hamstring graft diameter							
	Coefficient	*P* value						
Constant	.165	.870						
Age	2.606	.013						
Weight	− .003	.998						
Height	.141	.889						
BMI	.077	.939						
Abdominal girth	− .818	.418						
Thigh length	.270	.788						
Thigh circumference	− .120	.905						

## Discussion

The ability to predict the length of the hamstring graft pre-operatively is of great importance and may help the surgeon in the decision to achieve an acceptable diameter for the autograft in ACL reconstruction.

This study demonstrated a positive correlation between the patient height and thigh length with semitendinosus and gracilis graft length; this positive correlation was also reported in previous literatures [14–17].

In addition, we could not find a positive correlation between height and the final graft diameter; however, several studies have demonstrated such positive correlation among females [11–13, 18, 19]. In another study of 89 Asian males who underwent primary ACL reconstruction with quadrupled hamstring autograft also were unable to show this correlation [20].

Furthermore, we found no correlation between BMI and the final graft diameter in males as the females were excluded in our study. In previous studies, one author reported that BMI was correlated to the graft diameter in females but not in male [21], another author showed no correlation with BMI in both genders [11], and few authors had shown only a weak positive correlation between BMI and the quadrupled hamstring graft diameter [21, 22].

It is interesting to note that, in our study, the patient age was the only independent variable that had a positive correlation with the final graft diameter. The previous studies had demonstrated a negative correlation with age as a predictor of final quadrupled hamstring graft diameter [12, 13, 23].

Additionally, we were able to find a significant negative correlation between abdominal girth with gracilis and semitendinosus thickness as well as a significant positive correlation between thigh circumference and only gracilis thickness, which were not reported in previous literatures.

Some of the limitations of our study include small sample size, exclusion of females, the measurement technique is challenging and may be inaccurate for a thin tendon with varying width and thickness along the length and having two different surgeons involved in graft harvesting and measurement, possibility of other confounding factor and statistical artifacts to add more, and variability of amount of tension applied on the graft during sizing has an effect on the final graft caliber measurements intra-operatively.

## Conclusion

This study demonstrated that some anthropometric measurements had a positive correlation with the hamstring graft length and diameter in male patients. Hence, these results provide preliminary support for the use of some anthropometric measurements in the preoperative planning and prediction of the hamstring graft length and diameter in anterior cruciate ligament reconstruction.
